# Whole exome sequencing combined with dynamic ultrasound assessments for fetal skeletal dysplasias: 4 case reports

**DOI:** 10.1097/MD.0000000000031321

**Published:** 2022-10-28

**Authors:** Li Wang, Ruiqi Li, Jingfang Zhai, Bei Zhang, Jiebin Wu, Libo Pang, Ying Liu

**Affiliations:** a Xuzhou Clinical Medical School of Nanjing Medical University, Nanjing, Jiangsu, China; b The Second Clinical Medical School of Southern Medical University, Guangzhou, Guangdong, China; c Department of Prenatal Diagnosis Medical Center, Xuzhou Central Hospital, Xuzhou, Jiangsu, China.

**Keywords:** fetal skeletal dysplasias, multidisciplinary consultation, prenatal diagnosis, ultrasound examination, whole exome sequencing

## Abstract

Fetal skeletal anomalies are one of the most common and potentially pathogenic developmental abnormalities detected by ultrasound screening. Any suspected fetal skeletal dysplasias often require further comprehensive evaluations.

**Patient concerns:**

Here 4 families with adverse fetal skeletal system histories were enrolled, including their histories of gestation, childbirth, familial skeletal abnormalities, and pregnancy outcomes. The corresponding diagnosis were done by whole exome sequencing (WES) combined with dynamic examination.

**Diagnosis:**

All of the families were definitively diagnosed through cytogenetics, molecular genetics, ultrasound, combined with multidisciplinary evaluation. Both of the fetuses in case 1 and case 2 were diagnosed with thanatophoric dysplasia type I, while the neonate in case 3 was diagnosed with Apert syndrome and a 3-years-old proband daughter with Crouzon syndrome in case 4.

**Interventions:**

We conducted karyotyping, copy number variation sequencing (CNV-seq), combined with WES to evaluate genetic conditions of abnormal fetus, neonate or proband patient. WES was preferred to obtain a relatively definitive diagnosis.

**Outcomes:**

In cases 1 and 2, the families decided to choose termination of pregnancy due to fatal dysplasias. The couple in case 3, delivered a female baby diagnosed with Apert syndrome. Fortunately, in case 4, the family, which had a 3-years-old baby with Crouzon syndrome, gave birth to a healthy baby through prenatal diagnosis.

**Lessons subsections:**

Invasive prenatal diagnosis and dynamic assessments for the management of fetal skeletal dysplasias could contribute to revealing possible causes of fetal skeletal abnormalities and help clinicians conduct further genetic counseling in clinical practice.

## 1. Introduction

Fetal skeletal dysplasias are a kind of rare heterogeneous genetic disorders with a prevalence of 2.4 to 4.5 in 10,000 birth.^[1]^ It includes systemic congenital skeletal malformations and local limb malformations in the fetus. The skeletal dysplasias are divided into lethal skeletal dysplasias and non-lethal skeletal dysplasias. Thanatophoric dysplasia (TD) is the most common lethal skeletal type as an autosomal dominant inherited disease caused by the mutation of the gene fibroblast growth factor receptor 3 (FGFR3), with the incidence of 2 to 3 in 1,00,000 births, and most cases die in the perinatal period.^[2]^ The fetuses with TD in ultrasonography often show short limbs, narrow thorax, and macrocephaly with frontal bossing. According to the existence of a ``clover-leaf’’ skull and curved/ straight femurs, TD can be divided into 2 types, type I and type II Apert syndrome and Crouzon syndrome, are also autosomal dominant inherited syndromes, characterized by craniosynostosis, skull and facial deformities.^[3]^ Apert syndrome, known as acrocephalosyndactyly, has severe symmetrical syndactyly on hands or feet, with a prevalence of 1 in 65,000 births.^[4]^ It is due to the mutations of fibroblast growth factor receptor 2 (FGFR2) associated with paternal age.^[5]^ Crouzon syndrome is featured by acrocephaly, exophthalmos, hypertelorism, strabismus, and hypoplastic maxilla with normal hands and feet, which is often caused by FGFR2 or FGFR3.^[6]^

With the increasing development of ultrasound technology, most fetal skeletal structures can be identified by 12 weeks gestation. However ossification does not stop in the subsequent gestation and early fetal skeletal system abnormalities cannot completely indicate abnormal fetal development.^[7]^ Once ultrasound reveals suspected skeletal abnormal performance of a fetus, it is often an urgent challenge for clinicians to communicate with parents and provide them with necessary diagnostic methods and relatively reliable prognosis information. Hence, further diagnosis of early fetal skeletal abnormalities and dynamic follow-up should be acquired to judge possible fetal outcomes. In addition, to some extent, it should be emphasized that 1 primary task of prenatal diagnosis is to distinguish fatal fetal skeletal abnormalities presented.

Meanwhile, nowadays more and more molecular genetic approaches have been applied in prenatal diagnosis, many diseases can be diagnosed by whole exome sequencing (WES) based on next-generation sequencing techniques.^[8]^ Prenatal genetic evaluation can offer the parents more prospective information about the diagnosis, prognosis, and recurrence risk in future pregnancy to make informed decisions and better perinatal management.^[9]^

## 2. Case presentation

The 4 cases with adverse pregnancies or delivery histories of skeletal dysplasias were illustrated as the following. This study was approved by Xuzhou Central Hospital Ethics Committee (No. XZXY-LJ-20190210-037). All mutations with FGFR-related genes derived from de novo autosomal dominant inheritance. All the couples recruited in our cases signed the written consents for publication.

### 2.1. Case 1

**Figure 1.**
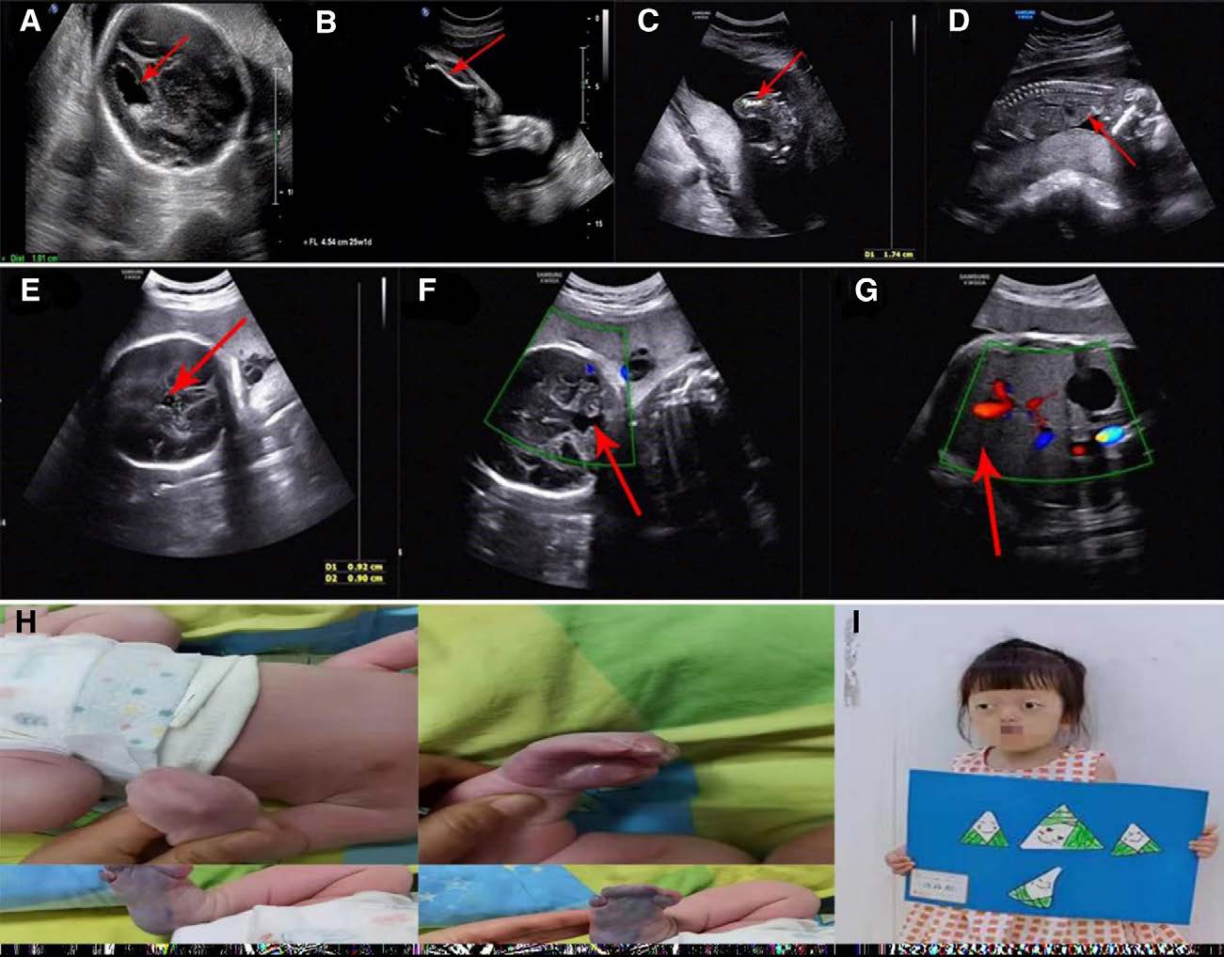
Clinical features of 4 cases. Case 1: (A) the mild dilation of the left ventricle (10.1 mm) in ultrasound; (B) “telephone receiver shape “curved femurs and the length of fetal femur was 4.54 cm at a gestation of 30 weeks. Case 2: (C) ultrasonography indicated short diameter lines of long bones (FL 17.4 mm) at 17w + 4d; (D) “bell-shaped” narrow thorax at 18 w + 4 d. Case 3: (E) intracranial cystic structure about 9.2*9.0 mm; (F) hypoplasia of cerebellar vermis; (G) indistinct gallbladder; (H) tight fusion of all fingers and the II-V toes symmetric syndactyly. Case 4: (I) appearances as Crouzon syndrome with skull and facial deformities, ocular hypertelorism, wide toes. FL = femur length.

**Figure 2.**
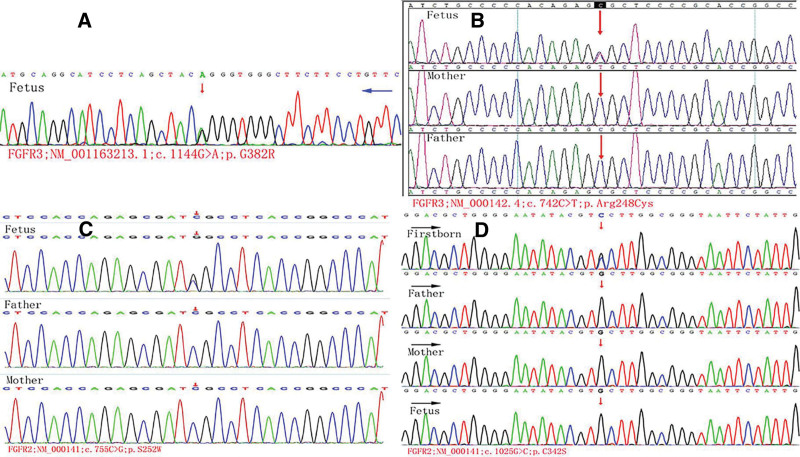
The WES results of 4 cases: Case 1: (A) a de novo mutation in FGFR3 (exon9:c.1144G > A:p.G382R) of the fetus; Case 2: (B) a de novo pathogenic mutation of the fetus in FGFR3 gene (c.742C > T); Case 3: (C) a de novo variant of FGFR2(c.755(exon7) C > G) of the newborn; Case 4: (D) the first proband daughter showed FGFR2 c.1025(exon8) G > C; p.C342S, and no mutation occurred in the fetus during this pregnancy. FGFR2 = fibroblast growth factor receptor 2, FGFR3 = fibroblast growth factor receptor 3, WES = whole exome sequencing.

A 39-years-old healthy woman, Gravida 2, Para 1 (G2P1), had routine antenatal care. Umbilical venipuncture was performed due to mild dilation of left ventricle (10.1 mm) (Fig. 1A) in ultrasound at 25 weeks gestation and both chromosome and copy number variation sequencing (CNV-se) (>100 Kb) were normal. Ultrasound showed both humerus and femurs were separated with head circumference at 30 weeks gestation. Both femurs (4.54 cm) were equivalent to 25 weeks gestation (Fig. 1B). The bone length of humerus and femurs in multiple ultrasounds deviated from the normal curve with gestational age gradually. The couple sought help from several hospitals in the following days. However, they rejected to conduct fetal WES. Finally, they terminated pregnancy at 31 + 5 weeks. Three months later, WES was performed on the fetal DNA and a mutated gene FGFR3 c.1144G > A was revealed (Fig. 2A) to define the fetal diagnoses with TD type I.

### 2.2. Case 2

A 25-years-old young woman, G2P0, once with an ectopic pregnancy in 2018, conceived naturally. At 17w + 4d gestational age (GA), ultrasound showed biparietal diameter (34mm) and femurs (17.4 mm) (equivalent to 15 weeks gestation) (Fig. 1C). The follow-up at 18w + 4d of gestation revealed fetal “telephone receiver” curved femurs and “bell-shaped” narrow thorax (Fig. 1D). Ultrasound at 19w + 4d of gestation indicated potentially lethal dwarfism. Finally, the couple decided to choose termination of pregnancy, and trio-WES was performed and a de novo mutation of c.742 C > T in FGFR3 was detected (Fig. 2B). The fetus was diagnosed with TD type I.

### 2.3. Case 3

The 34-years-old, G1P1, woman was referred due to fetal intracranial cystic structure of about 9.2*9.0mm (Fig. 1E), hypoplasia of cerebellar vermis (Fig. 1F), and indistinct gallbladder (Fig. 1G) at 23 weeks GA. Amniocentesis was conducted to confirm normal karyotype and CNV-se result. She routinely underwent prenatal care. Subsequently, a female baby, 4100 g and APGAR score 9/10, was delivered by cesarean at 39 weeks + 2 days. The neonate was transferred to pediatric intensive care unit due to tachypnea, peripheral cyanosis (Fig. 1H). And she was diagnosed with Apert syndrome of a variant of FGFR2 c.755 (exome 7) C > G by WES (Fig. 2C), characterized of high arched palate, nasal obstruction, frontal bossing, and syndactyly of fingers and toes. Three-dimensional reconstruction of trachea excluded malformations of the larynx and trachea. The absent cerebellar vermis suggested Dandy-Walker malformation, meanwhile, ventricular septal membrane defect and gallbladder absence were confirmed. The newborn finally died 1 month after birth due to respiratory insufficiency.

### 2.4. Case 4

The woman, 27-years-old, G2P1, has a 3-years-old daughter diagnosed with Crouzon syndrome (Fig. 1I). WES of the daughter showed FGFR2 c.1025 (exome 8) G > C; p.C342S (Fig. 2D). The couple accepted amniocentesis at a GA of 24 weeks + 3 days to conduct family WES. Fortunately, no mutation was detected in the fetus. Eventually, they gave birth to a healthy full-term female daughter.

## 3. Discussion

In this study, we talk about 4 categories of families at different stages of pregnancy with different reproductive choices, prognosis, and fetus outcomes. Our experience has been that the addition of WES to routine prenatal karyotype and CNV-Se can help diagnose the causes of these 4 fetal skeletal dysplasias. Moreover, the early joint efforts of a multidisciplinary team including clinical geneticists, prenatal diagnosticians, and ultrasound specialists, together with the strong cooperation of parents, may provide valuable clues for fetal diagnosis. Similarly, all of them once had adverse pregnancies or childbirth histories with skeletal dysplasias, which might bring great psychological harm to them.^[10,11]^ In the former 2 cases, due to the severity of dynamic ultrasonic abnormalities and fatal skeletal dysplasias (suspected TD type I), they chose termination of pregnancy, and the follow-up WES detection was performed after labor induction to further clarify the cause. In case 3, since there were no severe fetal anomalies detected on ultrasound, the clinicians didn’t make enough consideration about fetal monogenic hereditary disease of the skeletal system. Meanwhile, amniocentesis for karyotype analysis and CNV-se was normal, the couple decided to continue the pregnancy without further molecular and genetic examination. Although they encountered routine antenatal care and dynamic assessment, unfortunately, the family of case 3 delivered a child with Apert syndrome. This situation seems to be unavoidable in prenatal diagnosis, thus we should also pay more attention to the fetus with less severe fetal anomalies diagnosed by ultrasound. Further ultrasound follow-ups and comprehensive genetic evaluation should be carried out to find more information about the diagnosis, prognosis, and reproductive guidance, which can help parents make better reproductive choices and preparation physically and psychologically during pregnancy and after the birth of their child. The above same cause might have occurred in the Crouzon syndrome infant in the case 4 family. The couple in Case 4 received a prenatal diagnosis, avoided the same risk of adverse outcomes in the subsequent pregnancy, and had a healthy daughter.

WES detection showed FGFR2 in case 1 and Case 2, and FGFR3 in case 3 and case 4. Both FGFR2 and FGFR3 belong to the immunoglobulin-like receptor tyrosine kinases family. They are transmembrane receptors and are widely distributed on the surface of fibroblast-growth factors (FGF) target cells, which can mediate the biological activity of FGFs. FGFR2 plays an important role in the regulation of endochondral ossification. FGFR3 can regulate bone development by inducing bone morphogenetic protein type I receptor degradation. FGFR3 mutation could result in the impaired proliferation of growth plate chondrocytes and fetal clinical manifestations of limb shortening and premature joint closure.^[12]^ The above mechanism of FGFR2 and FGFR3 mutations fully explain the relevant clinical manifestations in the 4 cases. Prenatal fetal skeletal dysplasias can be detected effectively by ultrasound. The timeliness and severity of fetal ultrasound abnormalities might be quite important factors for prospective parents to make choice. In our study, similar skeletal manifestations with short femur length (FL) as the main phenotype were revealed at different times. The skeletal symptoms in case 2 appeared earlier than in case 1, and were accompanied by a more severe narrow thorax. As shown in the report, the couple in case 2 terminated the pregnancy in the 2^nd^ trimester, and the couple in case 1 in the third trimester. The later the termination, the more harmful it might be to the parents. Therefore, 1 of the strategies in the management of fetal skeletal dysplasias is to improve the accuracy and sensitivity of ultrasound diagnosis through molecular detection such as WES and CNV-Se for early pregnancy decisions. It is noted that WES has a high diagnostic value for prenatal skeletal dysplasias.^[13]^ In addition, short FL is a known sign of skeletal dysplasias, most of which may be related to monogenic abnormalities.^[14]^ Once short FL is revealed during the pregnancy, prenatal diagnosis using WES and CNV-se should be recommended for a definitive diagnosis to help parents make pregnancy decisions and know more about recurrence risk in future pregnancy. Meanwhile, parents’ future reproduction requirement may also influence their choices of the current tests for fetal abnormalities. In case 2, as the couple have further reproduction plans, they chose trio-WES which confirmed that the fetal genetic alteration was de novo, and the recurrence risk might be relatively low. However, the first family in case 1 opposed to parental WES verification due to no further reproduction requirements. In this report, we used karyotyping, CNV-se, combined with WES to evaluate genetic abnormalities. WES, especially WES of pedigree, should be recommended to be conducted in order to obtain a relatively definitive diagnosis and shorten the time required for diagnosis. We might strive for the consent and understanding of prospective parents as much as possible in the process.

In conclusion, invasive prenatal diagnosis combined with dynamic assessments for the management of fetal skeletal dysplasias could contribute to revealing possible causes of fetal skeletal abnormalities and help clinicians conduct further genetic counseling in clinical practice. Meanwhile, it is also important for parents with a history of skeletal dysplasias to receive psychological counseling in clinical practice, which could help them learn more about recurrence risk in future pregnancy.

## Author contributions

**Clinical consultation:** Jingfang Zhai and Jiebin Wu.

**Data collection:** Li Wang, Ruiqi Li

**Data curation:** Jingfang Zhai, Libo Pang, and Ying Liu.

**Supervision:** Jingfang Zhai,.

**Writing - original draft:** Li Wang, Ruiqi Li.

**Writing - review & editing:** Jingfang Zhai.

## Acknowledgments

We would like to thank all the couples for their participation in this study, the staff of the Department of Prenatal Diagnosis Medical Center Xuzhou Central Hospital, and the Berry Genomics Co. for the technical support for their contributions.
